# Polarity- and Sequence-Dependent Ionization of Therapeutic Antibody–siRNA Conjugates: Enabling Intact Multi-attribute Method for Comprehensive Characterization and Identity Release Assay

**DOI:** 10.1021/acs.analchem.5c06818

**Published:** 2026-02-27

**Authors:** Hao Liu, Jamie L. Veltri, P. Clayton Gough, Sean O. Crowe, Elizabathe Davis, Matt Whitaker, Ciaran Buckley, Zhirui Jerry Lian

**Affiliations:** † Bioproduct Research and Development, 1539 Eli Lilly and Company, Lilly Corporate Center, Indianapolis, Indiana 46285, United States; ‡ Eli Lilly Kinsale Limited, Dunderrow, Kinsale, P17 NY71 Co. Cork, Ireland

## Abstract

Antibody–siRNA conjugates (ARCs) represent a promising approach for delivering small interfering ribonucleic acids (siRNAs) to targeted cells, tissues, or organs. The complexity of the molecules poses great analytical challenges in identifying, characterizing, and monitoring their critical quality attributes (CQAs) during process development and manufacturing release. We developed a novel approach, intact multi-attribute method (iMAM), for ARC characterization using native size exclusion chromatography mass spectrometry (SEC-MS). The iMAM provides a simple and effective approach for monitoring CQAs such as identity, purity, higher molecular weight species (HMWS), lower molecular weight species (LMWS), N-glycosylation, modifications on unconjugated cysteine, linker hydrolysis, and drug-to-antibody ratio (DAR). The method was evaluated and qualified in a Good Manufacturing Practice (GMP) environment as an ARC drug substance (DS) and drug product (DP) identity release assay. During the method development, we found that ionization profiles of ARCs depended on the mass spectrometry operating polarity. Under positive polarity, more duplex siRNAs are preserved on the ARC molecules, whereas more ARC molecules with single-stranded RNA are present under negative polarity. Meanwhile, the presence of the antibody in ARCs provides a certain level of protection for the siRNA duplex during the ionization compared with siRNA alone. The ratio of the siRNA duplex on the ARC molecules during mass spectrometry detection was correlated with its GC content or melting temperature (*T*_m_). These findings provide a fundamental understanding of the relationship between siRNA and the antibody during ARC ionization and facilitate the development of effective methods for ARC characterization.

## Graphical abstract

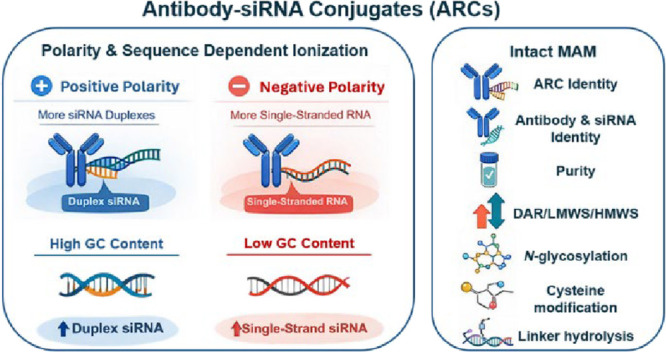

## Introduction

Since the pioneering work on small-interfering RNAs (siRNAs) by Tuschl in 2001, siRNA-based therapeutics have expanded significantly, with six products approved and numerous others in clinical trials.^1,2^ These therapies function by silencing target gene expression through mRNA degradation mediated by the RNA-induced silencing complex (RISC), thereby effectively and specifically inhibiting the synthesis of disease-associated proteins.^3^ Despite these therapeutic benefits, the clinical application of siRNA therapeutics faces several challenges, including the route of administration, vascular permeability limitations, systemic elimination, off-target effects, immune response, toxicity, and destruction by reticuloendothelial cells.^4^ To address these obstacles and enhance therapeutic efficacy, antibody–siRNA conjugates (ARCs) have been developed to deliver siRNA to cells, tissues, or organs specifically targeted by the antibody.^5–7^

ARCs combine large molecules, small molecules, and oligonucleotides in one entity. The physical and biochemical differences among these components pose significant challenges in developing effective analytical methods for their characterization. For example, siRNA is a highly negatively charged molecule, which often requires the use of amine-based ion-pairing agents (such as DIPEA or TEA) in the mobile phase to enable effective retention and separation on reverse-phase (RP) columns.^8^ In contrast, reverse-phase chromatography is routinely used for intact antibody analysis without such amine-based modifiers.^9^ For mass spectrometry (MS) analysis of antibodies and antibody–drug conjugates (ADCs), positive ionization mode is typically preferred, whereas siRNAs, due to their acidic and negatively charged nature, ionize more efficiently in the negative mode.^10,11^ Upon conjugation, the optimal ionization polarity of the ARCs becomes uncertain, requiring further evaluation. The guanine-cytosine (GC) content of siRNA significantly influences its melting temperature, thereby affecting the denaturation conditions required to dissociate the siRNA duplex.^12^ Although positive ionization has been applied in top-down ARC analysis,^13,14^ the impact of ionization conditions on siRNA duplex after conjugation has not been fully understood, especially with high temperature settings of the vaporizer and ion transfer tube, and different ionization polarities.

The MS-based multi-attribute method (MAM) has been extensively developed and adopted across the biopharmaceutical industry for the characterization of protein therapeutics.^15^ MAM aims to consolidate multiple conventional analytical assays into a single, comprehensive assay, offering novel insights that may not be accessible through traditional methods.^16^ With the advancement of antibody-drug conjugates, MS-based MAM characterization, at both the intact and subunit levels, has become an essential analytical approach. It enables confirmation of product identity and quantification of diverse conjugated species for precise control of the drug-to-antibody ratio (DAR), which are critical quality attributes for ensuring product consistency and efficacy.^11,17^ For ARCs, these quality attributes are equally critical for ensuring the product quality. However, a comprehensive assessment of these quality attributes often requires the application of distinct and complementary analytical techniques, as they cannot be fully captured by a single method. For example, anion-exchange chromatography (AEX) is primarily used to characterize the siRNA-to-antibody ratio (DAR), while size-exclusion chromatography (SEC) is widely utilized to monitor protein aggregation.^18,19^ The identification of DAR1 dimers and DAR2 species is particularly challenging due to the limited resolution of conventional analytical methods, which often lack the sensitivity and specificity required to distinguish closely related conjugation variants. There is a growing demand for the development of MS-based multi-attribute methods (MAM) tailored specifically for antibody-related conjugates, to enable comprehensive and streamlined characterization of their critical quality attributes.

The native SEC-MS method has been widely reported for characterizing therapeutic antibodies and antibody-drug conjugates at the intact protein level.^19,20^ The native SEC-MS facilitates the size-based separation and structural characterization of biotherapeutics under nondenaturing conditions. This approach preserves higher-order structures and noncovalent interactions, enabling comprehensive analysis of CQAs essential for biotherapeutic development and regulatory compliance.^21^ Moreover, the nonretentive chromatographic nature of SEC enables it to serve as a universal platform method for the analysis of diverse biotherapeutic modalities, including antibody, siRNA, and ARC, without specific interactions with the stationary phase.^22^ Thus, native SEC-MS is preferred as an identification method for ARCs.

In this study, we developed an iMAM method for ARC characterization using a native SEC-MS method. The method development was first focused on optimizing the mobile phase salt concentration to enhance both chromatographic separation and ionization efficiency. Ionization polarity was subsequently evaluated to refine the detection specificity. Method suitability was demonstrated using four distinct ARCs, each conjugated with different siRNA sequences with similar molecular weights. The MAM capability was assessed for the simultaneous evaluation of several critical quality attributes, such as identity, purity, HMWS, and LMWS, N-glycosylation, modifications on unconjugated cysteine, linker hydrolysis, and DAR. Finally, the method was validated in two Good Manufacturing Practice (GMP)-compliant laboratories and implemented as an identity assay for ARC lot release.

### Materials

ARC-1, ARC-2, ARC-3, ARC-4, ARC-5, the SS conjugate, and siRNA-linker-1 were generated at Eli Lilly and Company. An accelerated degradation study was performed with the ARC-2 molecule under stress conditions of 40 °C for 4 weeks. Ammonium acetate was purchased from Honeywell (Muskegon, MI), and water (Optima LC/MS grade) was obtained from Thermo Fisher Scientific (Waltham, MA). The theoretical melting temperature was calculated by SnapGene (Boston, MA).

### Native SEC-MS Method

Native SEC-MS was performed on a Thermo Exploris 240 Orbitrap mass spectrometer (Thermo Scientific, CA) with a HESI source coupled with a Thermo Vanquish UPLC (Thermo Scientific, CA). ARCs were separated on a BEH SEC column (4.6 × 300 mm, 1.7 μm, ACQUITY UPLC Protein BEH, Waters) with a flow rate of 0.2 mL/min using an isocratic mobile phase of 50 mM ammonium acetate at room temperature. The eluent was detected by a UV detector with a wavelength of 260 nm and then analyzed by the mass spectrometer with both positive and negative ion modes enabled. Ion source parameters were set as follows: ion transfer tube temperature at 275 °C, vaporizer temperature at 250 °C, and ESI voltage at 3800 V. The full spectrum was acquired with a range of 2500–8000 *m*/*z* for the conjugates and 700–7500 *m*/*z* for the extended MS spectrum to collect both siRNA- and ARC-related species at a resolution of 30000 and a normalized automatic gain control (AGC) (%) of 300. Thermo Chromeleon software (Thermo Scientific, CA) was used for intact deconvolution, while Thermo Biopharma Finder (Thermo Scientific, CA) was used for siRNA sequencing. The optimized MS settings for ARC-1 under a positive polarity are as follows. The flow of sheath gas was lowered from 25 to 20, while the flow of auxiliary gas was lowered from 10 to 5. The temperature of the vaporizer was lowered from 250 to 150 °C with the same ion transfer tube temperature at 275 °C.

## Results and Discussion

### Native SEC-MS Method Development

Each ARC (antibody–RNA conjugate) in our study is built on a “one-arm” antibody scaffold (Figure 1). This one-arm antibody consists of one light chain (LC), one full heavy chain (HC), and a second heavy chain that is truncated to only the Fc region; these subunits are covalently linked by the usual interchain disulfide bonds (one LC–HC disulfide and two HC–Fc disulfides at the hinge). The siRNA sense strand, covalently conjugated with a chemical linker, is further covalently attached to an exposed cysteine on the antibody via maleimide–thiol conjugation chemistry. In contrast, the antisense strand is not covalently attached; it binds the sense strand by Watson–Crick base pairing, forming a duplex through noncovalent interactions. Native SEC-MS can preserve noncovalent interactions, providing great advantages for ARC characterization. Mobile phases with volatile salts, such as ammonia acetate, are often used for developing native SEC-MS methods. When coupled with a mass spectrometer, the concentration of the ammonium acetate greatly affects separation and the ionization efficiency. Low ammonium acetate concentration provides better ionization, while high concentration might provide better separation on an SEC column. To figure out the separation resolution and MS ionization, individual scaffolds (DAR0), DAR1, and DAR2 of ARC-4 molecules were prepared and analyzed with 50 mM ammonium acetate. The overlay chromatograms of the UV trace (Figure 2) show that these DAR species can be effectively separated by 50 mM ammonium acetate with good MS ionization. Therefore, 50 mM ammonium acetate was used for our native SEC-MS. This highlights the utility of native SEC-MS for accurate DAR characterization and in-process monitoring of conjugation profiles without the need for sample preparation.

**Figure 1:**
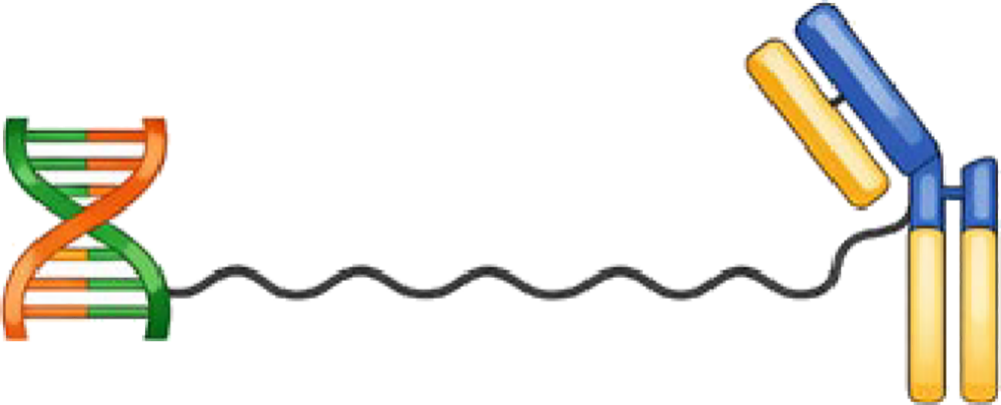
The schematic figure of the ARC used in this study. The sense strand is covalently linked with a linker, and the sense strand-linker together is further conjugated with the one-arm antibody via a cysteine-maleimide reaction. The one-arm antibody consists of one light chain, one full-length heavy chain, and one truncated heavy chain with only the fragment crystallizable region and is covalently associated with interchain disulfide bonds. The antisense strand forms an siRNA duplex with the sense strand through noncovalent interactions. The conjugate site does not represent the exact conjugation site used in this study and does not impact the ionization observed. The schematic figure was an AI-generated image created by Lilly AI Image Generator.

**Figure 2:**
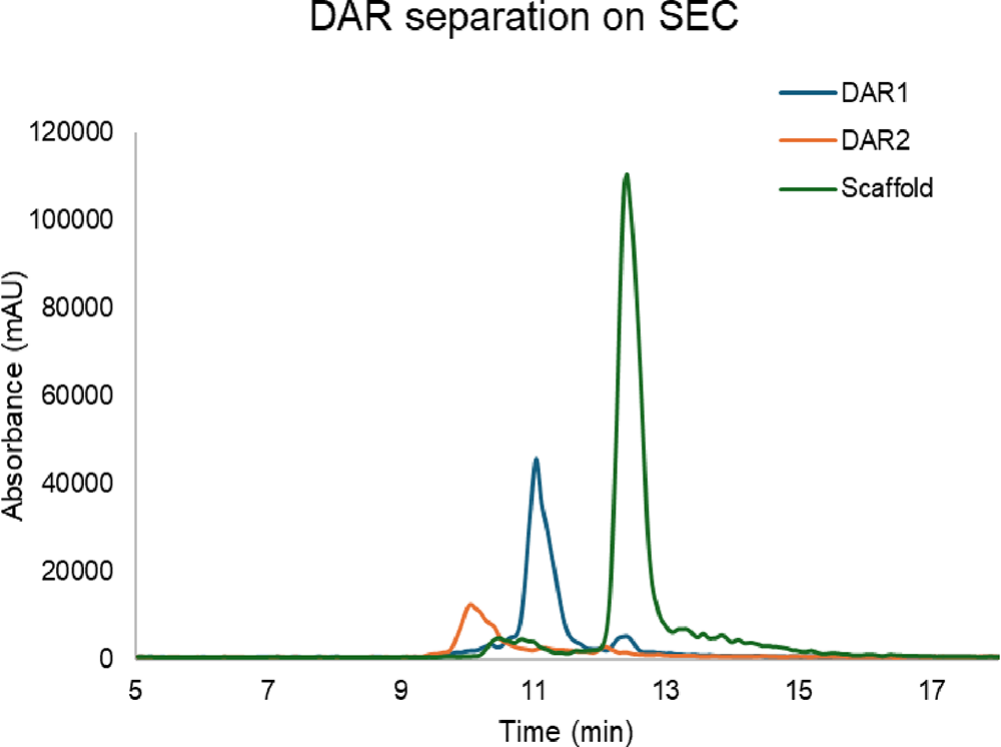
The separation of various DAR species on SEC-UV-MS. Each DAR species was injected on SEC, respectively, and the DAR separation is shown in the overlay UV chromatogram.

### Observations on ARC Ionization

Antibodies are typically ionized in positive mode during MS analysis, whereas siRNAs are usually ionized in negative mode. To find the best ionization parameters for ARCs where the two different moieties are conjugated together, we investigated the ionization of ARC molecules under both positive and negative polarity. Interestingly, we found that the polarity affects the ionization of the ARC molecules in our native SEC-MS method. As shown in Figure 3C,D, the most dominant component of ARC-2 detected was the intact molecule (Intact MW), i.e., antibody with siRNA duplex, under positive polarity, whereas the most abundant under negative polarity was the intact antibody with SS only, i.e., loss of the antisense strand (Intact MW-AS). This can be explained by the nature of the antibody, siRNA, and the HESI process.^23,24^ During positive ionization, the inherent negative charges on the siRNA duplex were neutralized by an excess amount of positive charge on the droplets generated in the positive electric field. This could minimize the net charge and keep the noncovalent siRNA duplex together. Conversely, both the antibody and siRNA duplex were negatively charged under negative polarity. The overall increased net negative charge results in strong electrostatic repulsion, accelerating the dissociation of siRNA duplex. As a result, more intact ARC with the siRNA duplex was observed in positive polarity (Figure 3C,E,G) and at a relatively lower abundance in the negative mode (Figure 3D,F,H).

**Figure 3:**
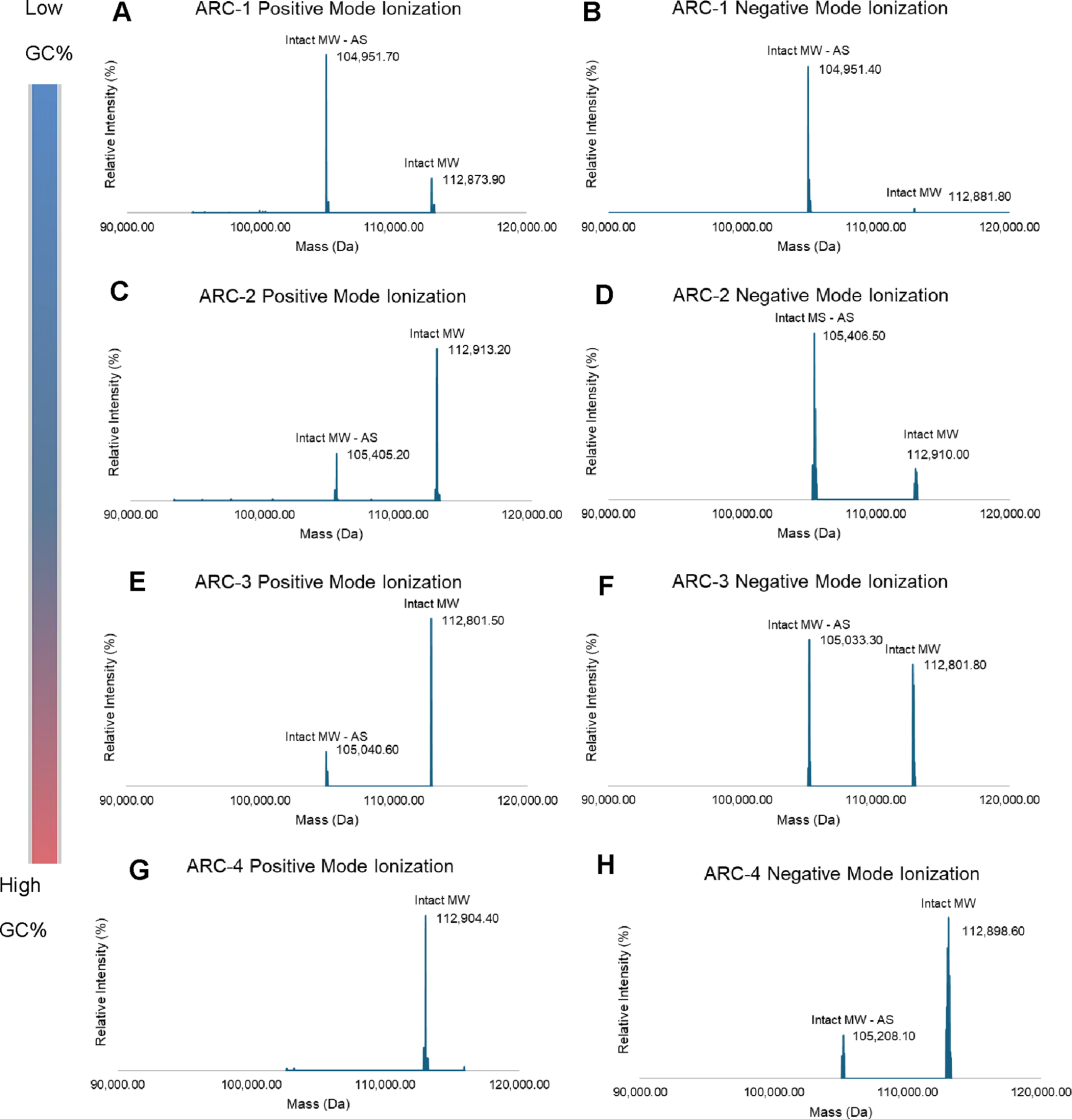
Deconvoluted MS spectra of 4 different ARCs with different siRNA sequences under positive and negative polarity. Intact with siRNA duplex was mainly observed under positive polarity, while the intact with the loss of AS was more under negative polarity. The higher percentage of GC in AS, the more intact siRNA duplex was observed under both polarities. The percentage of GC in AS is in the following order: ARC-1 < ARC-2 < ARC-3 < ARC-4.

More interestingly, the siRNA duplex used in ARC-4 was fully dissociated in negative polarity (Figure S1), whereas most of the duplexes in ARC-4 remained intact after conjugation under the same ionization conditions (Figure 3H). It suggests that the antibody in ARC could stabilize the siRNA duplex in negative ionization. This stabilization could be attributed to proton-transfer ion/ion reactions.^25^ Clearly, the electrostatic interaction between the antibody and siRNA under different polarities plays a role in the ARC ionization.

In addition, no predominance of intact ARC-1 was observed under positive polarity (Figure 3A), whereas under negative polarity, no predominance associated with AS loss was observed in ARC-4 (Figure 3H). As various ARCs were conjugated with different siRNA sequences in this study, it suggests that the sequence of siRNA also greatly affected the ARC ionization. Typically, the siRNA duplex with a lower GC ratio has a lower melting temperature (*T*_m_), indicating that less energy is required for dissociation. We plotted the relationship between either the GC ratio of one strand or the theoretical melting temperature and the percentage of intact molecules under both polarities (Table S1). As shown in Figure 4, the GC ratio is well correlated to the ratio of intact detected with *R* ^2^ values of 0.8763 and 0.9394 for positive polarity and negative polarity, respectively. The correlation between the theoretical melting temperature and the ratio of intact detected is also close to linear, with an *R* ^2^ of 0.8741 and 0.9081 for positive polarity and negative polarity, respectively. A similar linear algorithm has been widely used to calculate the melting temperature according to the Poland–Scheraga model.^26,27^ But modifications of the ribonuclease ring, such as 2′-fluorination (2′-F) or 2′-*O*-methylation (2′-*O*-Methyl), also impact the melting temperature, deviating from the linearity observed under both polarities.^28^ Similarly, the melting temperature was reported to impact the SEC analysis of divalent small interfering RNA.^29^ Our study is the first to observe the impact of siRNA sequence on ARC ionization during LC-MS analysis. This highlights the need to optimize the MS method based on the specific siRNA sequence of ARC. Moreover, the GC ratio in this study covered the range for most siRNA designs, roughly from 20% to 50%.^30,31^ Thus, the observed correlation can provide general guidelines for ARC LC-MS method development.

**Figure 4:**
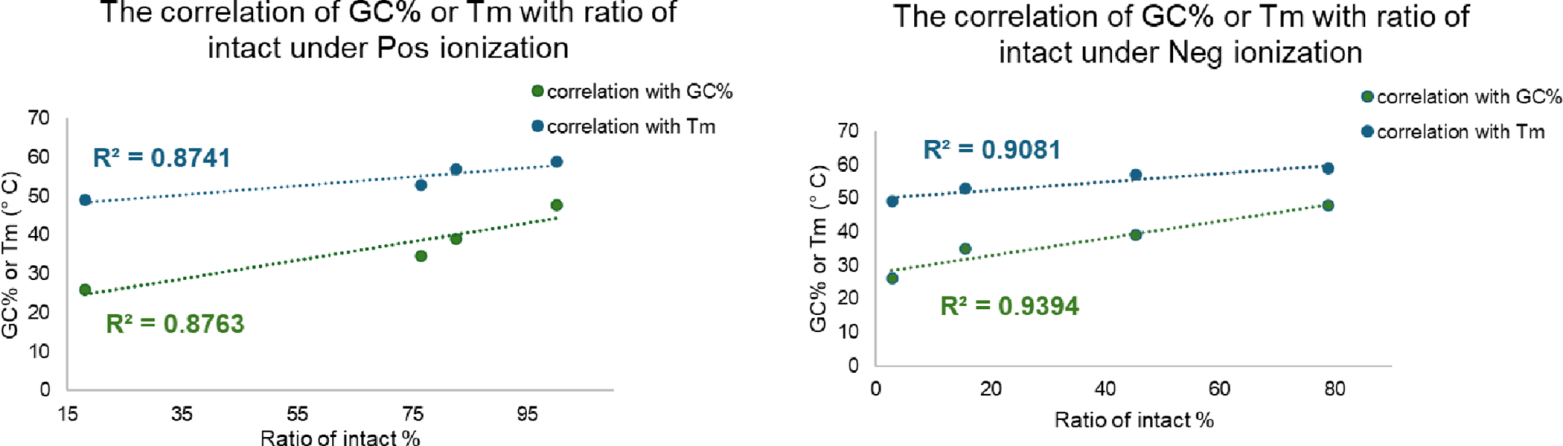
The correlation between GC% or *T*_m_ and the ratio of intact detected under positive and negative polarities is shown. The linearity from both polarities suggested that more intact ARC with the siRNA duplex would be detected with either higher GC% or higher melting temperature.

### Native SEC-MS Method as an Identity Method

Identity testing is required for all drug substances and drug products of protein therapeutics by regulators around the world, per the ICH guideline, to ensure efficacy and safety.^32^ The traditional identity method usually compares the retention time with a standard to confirm the identity, but might lack specificity for new modalities like ARC.^33^ To evaluate the suitability of the native SEC-MS method for identity testing, we tested four in-house ARC molecules. As previously noted, four ARCs ionized differently under positive and negative polarity (Figure 3). Additionally, for ARCs having dissociated AS during negative ionization, both ions of AS and those of intact ARC, with the loss of AS, were collected in one extended MS spectrum (Figure 5), and both measured masses after deconvolution matched with the theoretical masses within 100 ppm, which is the generally acceptable criterion for intact LC-MS analysis.^34,35^ Furthermore, the method provides an additional level of specificity by achieving 100% sequence coverage of the free AS detected in the extended MS spectrum (Figure 9B). As a result, our identity method is a two-factor identity method. The first factor is based on correct mass measurement of all components, including intact loss of AS, intact, and/or AS (intact mass and/or signature sequencing ions), while the second factor is based on signature ARC ionization behavior observed under positive and negative polarity and the influence of the siRNA sequence. These two factors provide additional assurance of the specificity of the identity method.

**Figure 5:**
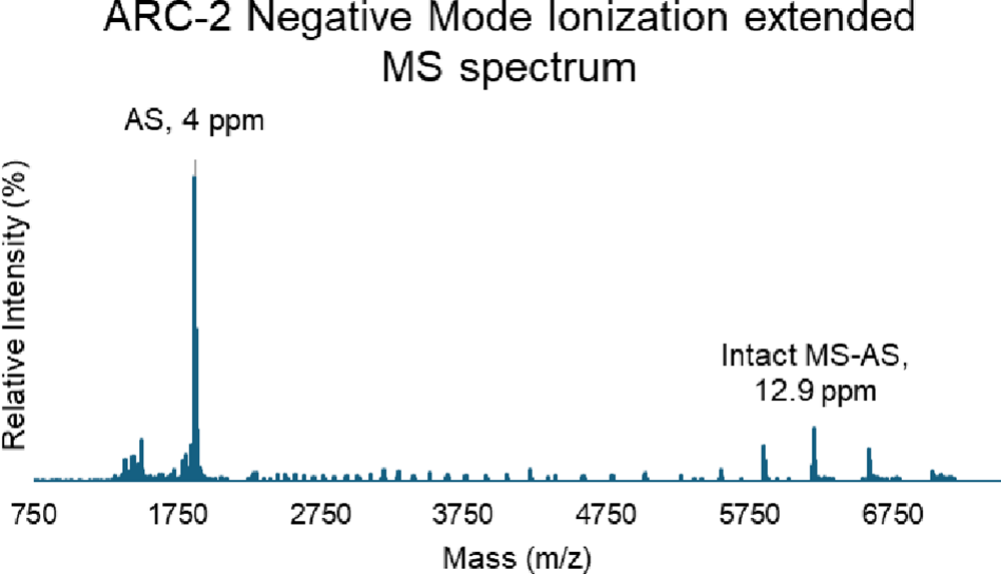
The extended MS spectrum of ARC-2 in negative mode. Both ions of AS and those of intact with the loss of AS were collected in one extended MS spectrum. The deconvolution of AS was based on the detected monoisotopic ions with a 4 ppm mass difference, while the deconvolution of intact ARC with the loss of AS was based on the ReSpectTM approach for isotopically unresolved ions with a 12.9 ppm mass difference.

### Native SEC-MS Method as an iMAM Method

SEC is the standard method to monitor aggregation and fragmentation of monoclonal antibodies in the biopharmaceutical industry. In this study, we compared the chromatographic performance of conventional analytical SEC with MS-compatible SEC. Separation using ammonium acetate in MS analysis was comparable to that obtained with phosphate and sodium chloride in analytical SEC, as shown in Figure 6A. In addition, integration at 260 nm in SEC-MS closely matched the results acquired at 214 nm in analytical SEC for both control and heat-stressed samples (Figure 6B). These findings demonstrated that MS-based SEC was relatively comparable in detecting aggregation and fragmentation to conventional analytical SEC.

**Figure 6:**
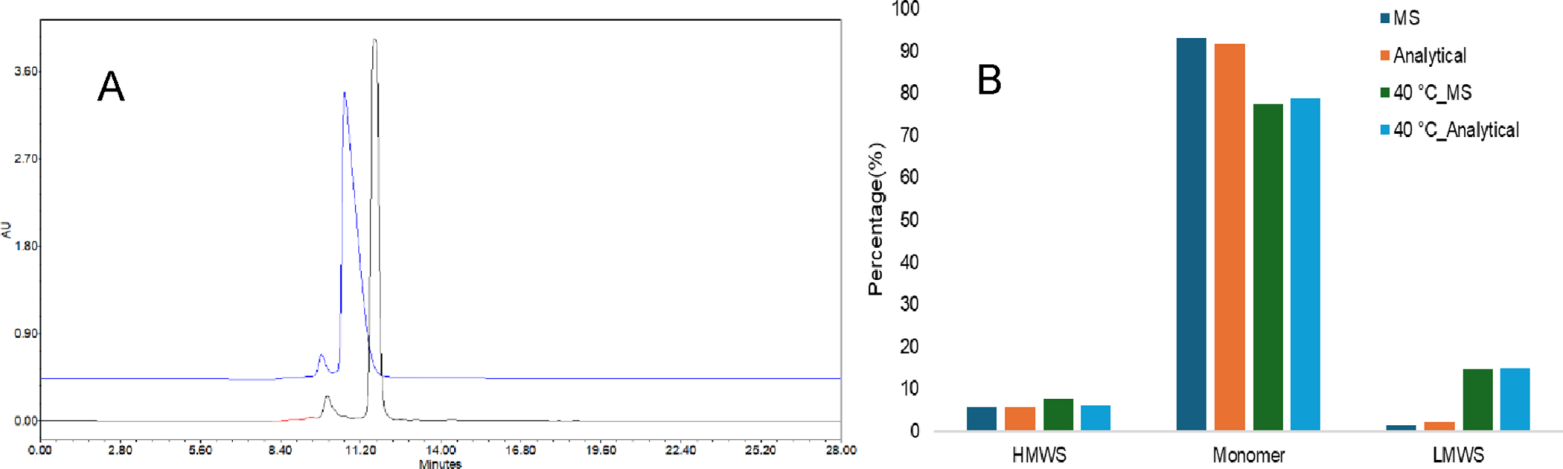
SEC UV data comparison between analytical SEC and MS-compatible SEC. A similar SEC separation was shown in A, the overlay UV chromatogram under 260 nm with ammonium acetate (used in MS SEC in blue) and with phosphate and sodium chloride (used in analytical SEC in black). A similar purity profile was also observed, as shown in B, by comparing the percentage of higher molecular weight species (HMWS), monomer, and lower molecular weight species (LMWS) of both control and heat-stressed samples using the analytical SEC with detection at 214 nm and the MS-compatible SEC with detection at 260 nm.

Conjugation was typically achieved through either engineered cysteines or free cysteines generated by the reduction of interchain disulfide bonds, likely introducing unconjugated cysteines. Characterizing modifications on these unconjugated cysteines is critical, as their presence can significantly impact the product quality. Using SEC-MS, such modifications can be identified. As illustrated in Figure 7A, cysteinylation was observed along with diverse N-glycosylation patterns. The hydrolyzed maleimide linker with +18 Da was also observed in the heat-stressed sample with the high-resolution MS instrument in Figure 7B. Monitoring linker hydrolysis enables enhanced quality control of DS/DP across various matrixes and storage conditions. Overall, the SEC-MS approach enables simultaneous monitoring of unconjugated cysteine modifications, linker hydrolysis, and N-glycosylation profiles.

**Figure 7:**
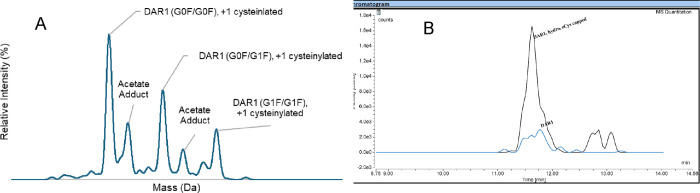
Characterization of eCys modification, N-glycosylation, and linker hydrolysis of DAR1 ARC. (A) shows the modification of unconjugated cysteine along with various N-glycosylation patterns observed in the deconvoluted mass spectrum. (B) represents the EICs of two components of the DAR1 heat-stressed sample. One is DAR1 with the linker hydrolyzed and the unconjugated cysteine cysteinylated (black trace), and the other is DAR1 (blue trace). The observed masses match the theoretical masses (≤100 ppm).

Effective gene silencing requires proper incorporation of siRNA, especially the AS, into the RISC. The dissociation of the siRNA duplex is therefore a critical quality attribute in ARC characterization. We observed that some siRNA duplexes were easily dissociated under negative ionization, raising the question of whether this dissociation occurs in the actual product or is an artifact of MS analysis. We addressed the concern in two ways. (i) Optimization of MS conditions: We adjusted the MS source parameters to minimize any in-source dissociation of the siRNA duplex. In particular, we found that using optimized positive-ion mode settings (e.g., reduced vaporizer temperature and gas flows) eliminated detectable strand loss for the ARC-1 conjugate, which has the lowest duplex melting temperature (GC content of 26%) in our set. Under these gentler conditions, ARC-1 showed no dissociation of the AS strand during analysis (Figure 8). Since most siRNA sequences have GC contents between ∼20% and 50%, these settings should broadly prevent artifactual duplex dissociation in our SEC–MS method. (ii) Spike-in study and SEC separation: We also performed the spike-in recovery experiment to demonstrate the method’s ability to detect real free strands. We spiked a known free antisense strand into a sample containing only the sense strand–linker–antibody conjugate (SS conjugate). Under our native SEC–MS conditions, the spiked AS found its complementary sense strand and formed a duplex (essentially reforming the intact ARC in solution), which has the same retention time as the intact ARC in Figure 9A. In our experiment, a free AS that remained single-stranded eluted at ∼16 min (detected at 260 nm)—clearly later than the main ARC peak. Moreover, the ARC with only SS exhibited a slightly later elution on the SEC column due to its lower molecular weight (Figure 9A). By extending the MS detection range, both the free AS strand and the intact ARC without the AS strand were accurately detected within a single spectrum, and the free AS strand was further fully sequenced (Figure 9B). Thus, the method can distinguish real free single-strand impurities from any artifact. In summary, by combining SEC separation (which ensures that any free SS/AS impurities appear at different retention times) with optimized MS settings (to avoid artificial dissociation), we can reliably detect and quantify genuine single-strand impurities.

**Figure 8:**
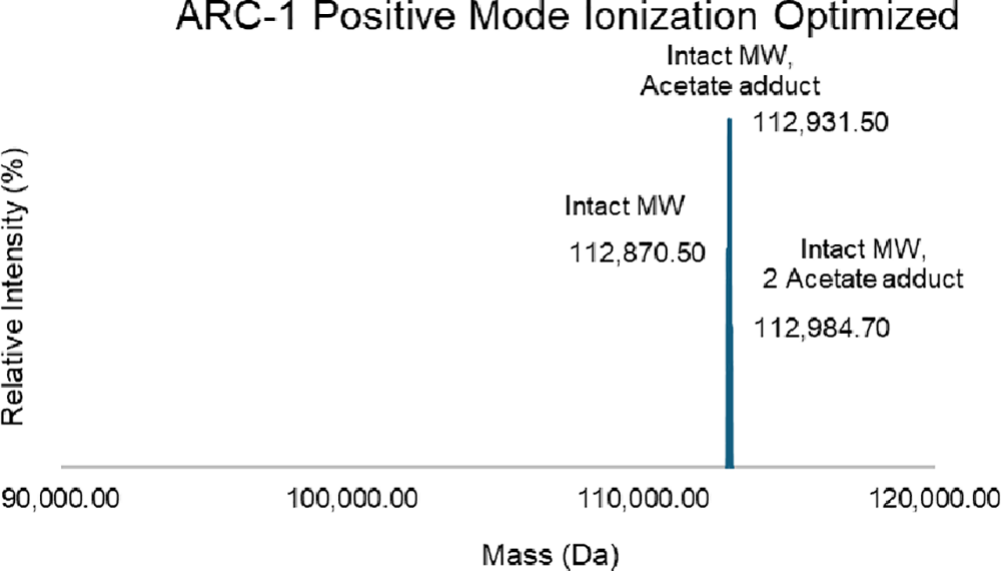
Minimize free siRNA-related impurities from method artifacts. With the gentle LC-MS conditions, there is no AS detected. The method artifact could be efficiently prevented.

**Figure 9:**
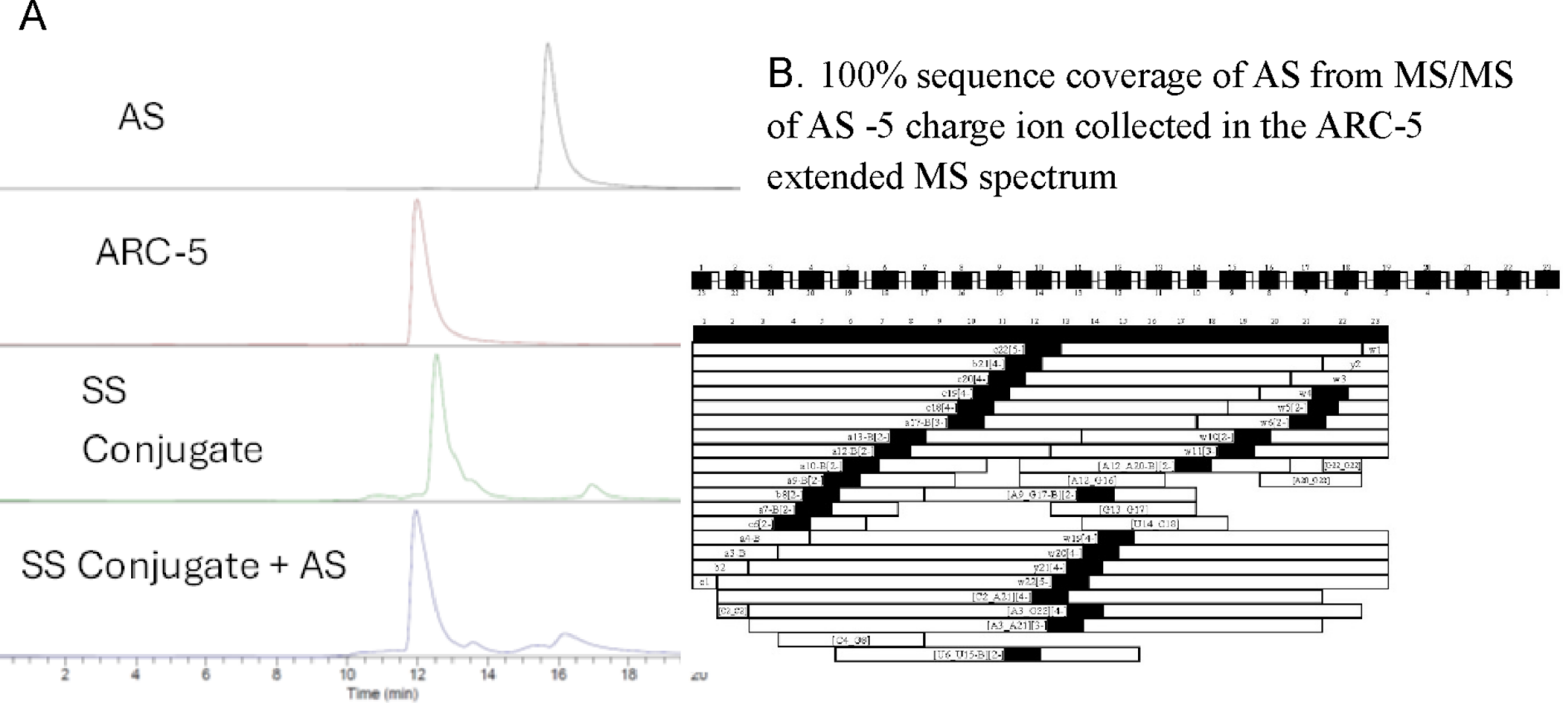
Distinguish free siRNA-related impurities by SEC-MS and SEC-MS/MS. (A) The overlay UV chromatogram at 260 nm reveals that the free antisense strand exhibits a distinct retention time compared to ARC-related species, confirming the method’s capability to accurately identify free siRNA impurities. (B) The sequence coverage of AS is 100% from MS/MS of AS (the −5 charge ion) collected in the ARC-5 extended MS spectrum.

Furthermore, the identity of in-house siRNA-linker-1 was successfully confirmed with the same method (Figure 10). As molecular weight enables more specific identification than relative retention time and does not depend on critical reagents such as ion-pairing agents commonly used for siRNA release testing, this approach minimizes risks typically associated with method transfer in conventional analytical workflows. Measuring the molecular weight also provides better quality control of linker hydrolysis in the intermediate, which will not be conjugatable and will impact the overall conjugation yield. Moreover, the native SEC-MS method minimizes the use of organic solvents in the conventional ion-pairing RPLC method for oligonucleotide identification, promoting environmental sustainability. With 100% sequence coverage of AS in the ARC-5 extended MS spectrum, the method can identify siRNA based on both intact mass and signature sequencing ions from MS2 sequencing, as shown in Figure 9B. The ability to measure free siRNA-linker further enables monitoring of its release via retro-Michael addition, thereby enhancing the safety assurance of ARC DS/DP.

**Figure 10:**
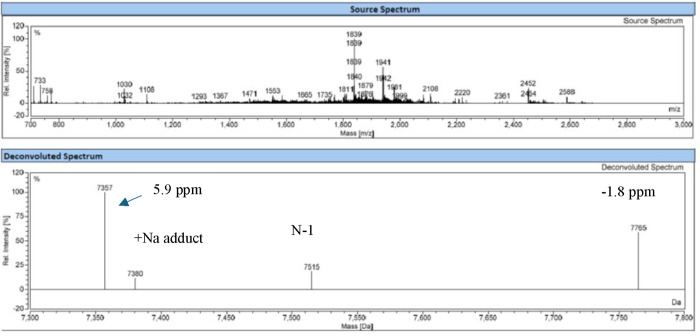
Both ions of the two strands of siRNA-linker-1 were detected in one MS spectrum. The deconvolution was based on the detected monoisotopic ions with −1.8 and 5.9 ppm mass differences, respectively.

Overall, the iMAM method of native SEC-MS can characterize more than 6 CQAs during the process development and product release at the intact level (Figure 11). Both the identity of ARC and the siRNA-linker intermediate can be confirmed by one platform method, which can be further expanded to confirm the identity of the antibody intermediate. Usually, analytical AEX is preferred for DAR profiling, and analytical SEC is selected for ARC size variant analysis. The iMAM method can not only merge those two analytical methods into one simple analysis to speed up the development process, but also differentiate DAR2 from the DAR1 dimer to ensure the product safety. Additionally, with both UV and MS capabilities of the iMAM method, it addresses in-depth characterization needs, including characterizing modifications of unconjugated cysteine or linker, N-glycosylation, and siRNA dissociation.

**Figure 11:**
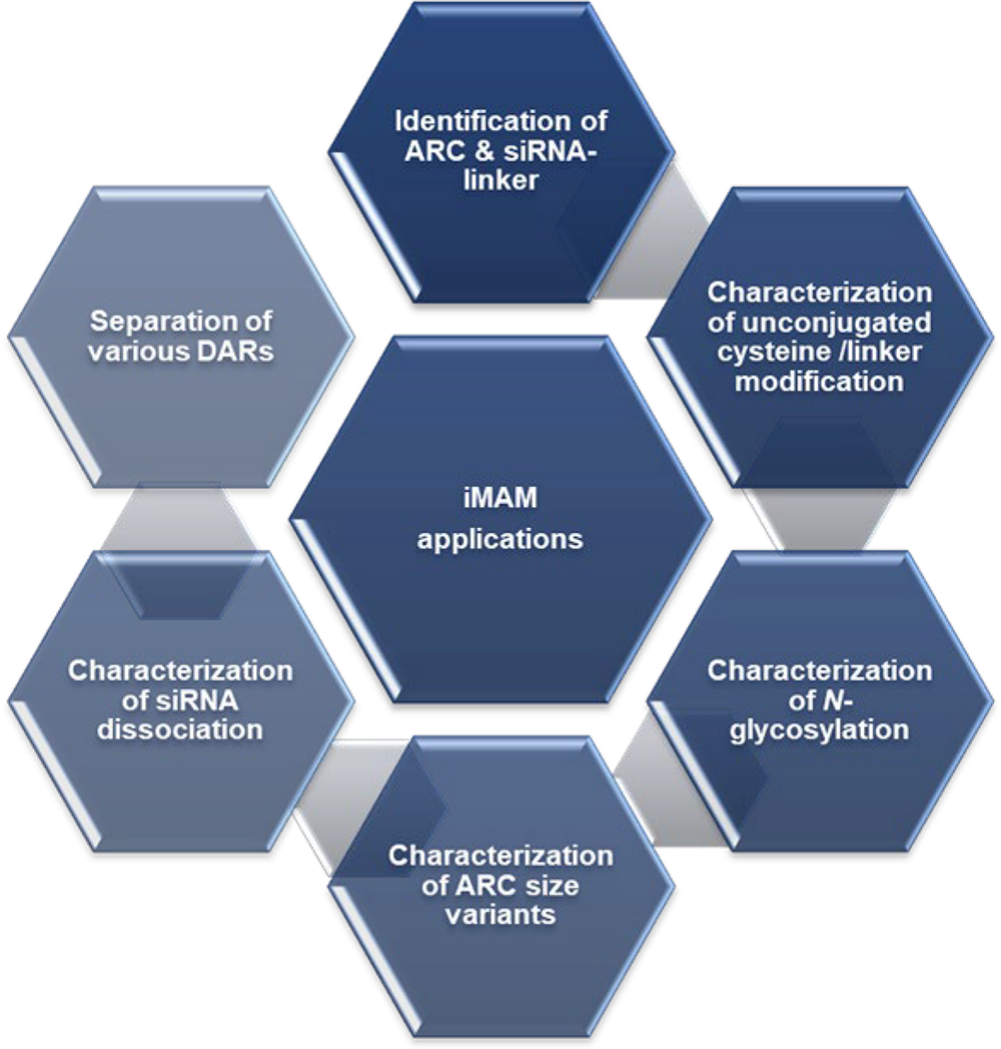
Summary of the iMAM method. In a single analysis, the iMAM method is capable of identifying ARC (based on molecular weight and signature ionization behavior), identifying siRNA-linker (based on monoisotopic molecular weight and/or signature sequencing ions), characterizing unconjugated cysteine or linker modification, characterizing N-glycosylation profile, characterizing ARC size variants, characterizing siRNA duplex dissociation, and separating various DARs.

### Validation of the iMAM Method in the GMP Environment

To implement the iMAM method as an identity method, specificity, repeatability, and solution stability were evaluated in two GMP laboratories (Table S2). Triplicate preparations of the drug substance yielded consistent chromatograms, clearly distinguishable from those of the matrix (Figure 12). The measured masses from the two GMP laboratories exhibited good repeatability (RSD < 1%) and good stability at 5 °C for 3 days. The iMAM method has been successfully validated in two GMP laboratories, demonstrating robust specificity, repeatability, and solution stability. Accordingly, the iMAM method is qualified for the release testing of ARC drug substance and drug product batches.

**Figure 12:**
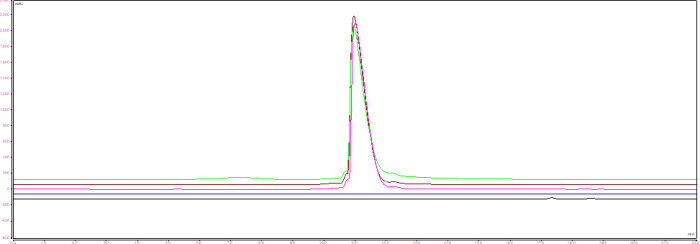
Overlay SEC-UV chromatograms of ARC-4 DS from triplicate preparations, along with water and solvent matrix.

## Conclusions

In this study, we developed an iMAM method to confirm the identity and characterize ARC (Figure 1) during process development and product release. It was first observed that ARC ionization was different under positive and negative polarity. The duplex of siRNA on ARC tends to remain intact under positive polarity, even with a high source temperature above its melting temperature, but is more easily dissociated under negative polarity. Moreover, the dissociation of siRNA duplex under negative polarity is highly correlated with its GC ratio or melting temperature, suggesting a signature ionization behavior related to the siRNA sequence. Combining the signature ionization behavior of ARC under negative polarity with the accurate mass measurement of each component (intact ARC, ARC-AS, and/or AS (intact mass and/or signature sequencing ions)), our ID method enables significant specificity for ARCs. In addition to confirming the identity of ARC, the iMAM method can also serve as a platform method to confirm the identity of both the siRNA-linker intermediate and the antibody intermediate. The iMAM method can simultaneously monitor multiple CQAs, including unconjugated cysteine or linker modifications, N-glycosylation, ARC size variants (HMWS, LMWS, monomer purity), siRNA dissociation, and DAR profiling in a single analysis. Finally, the iMAM method has been validated as an identity release assay in two GMP laboratories, demonstrating its feasibility for global implementation across GMP testing sites handling complex new modalities.

## Supplementary Material



## Notes

Figure S1: The deconvoluted spectrum siRNA-linker intermediate of ARC-4 in negative mode; Table S1: The correlation between the GC ratio or theoretical melting temperature and the ratio of intact detected under both polarities for all 4 ARCs. Table S2. The qualification parameters evaluated in GMP environment for identity release assay (PDF)
