# Supplementary material for: Polarity- and Sequence-Dependent Ionization of Therapeutic Antibody–siRNA Conjugates: Enabling Intact Multi-attribute Method for Comprehensive Characterization and Identity Release Assay
Source: Anal Chem. 2026 Feb 27;98(10):7422–31. doi: 10.1021/acs.analchem.5c06818 (PMC13000873; doi:10.1021/acs.analchem.5c06818)
Supplement: Supplementary file 1 [file ac5c06818_si_001.pdf]

Supporting Information

Polarity and Sequence-dependent ionization of therapeutic antibody-siRNA conjugates: enabling intact multi-attribute method (iMAM) for comprehensive characterization and identity release assay

Hao Liu [1,] *, Jamie L Veltri [1], P Clayton Gough [1], Sean O Crowe [1], Elizabathe Davis [1], Matt Whitaker [1], Ciaran Buckley [2], Zhirui Jerry Lian [1], *

1. Bioproduct Research and Development, Eli Lilly and Company, Lilly Corporate Center, Indianapolis, Indiana 46285, United States.

2. Eli Lilly Kinsale Limited, Dunderrow, Kinsale, Co. Cork, P17 NY71, Ireland

*Corresponding author.

Table of Content

Content	Page No.
Figure S1. The deconvoluted spectrum siRNA-linker intermediate of ARC-4 in negative mode.	S2
Table S1. The correlation between the GC ratio or theoretical melting temperature and the ratio of intact detected under both polarities for all 4 ARCs.	S3
Table S2. The qualification parameters evaluated in GMP environment for identity release assay.	S4

Figure S1. The deconvoluted spectrum siRNA-linker intermediate of ARC-4 in negative mode. The siRNA duplex was dissociated and detected as two species (7367 Da and 7809 Da, separately) whereas the duplex was associated together when analyzing ARC-4 under the same MS conditions.

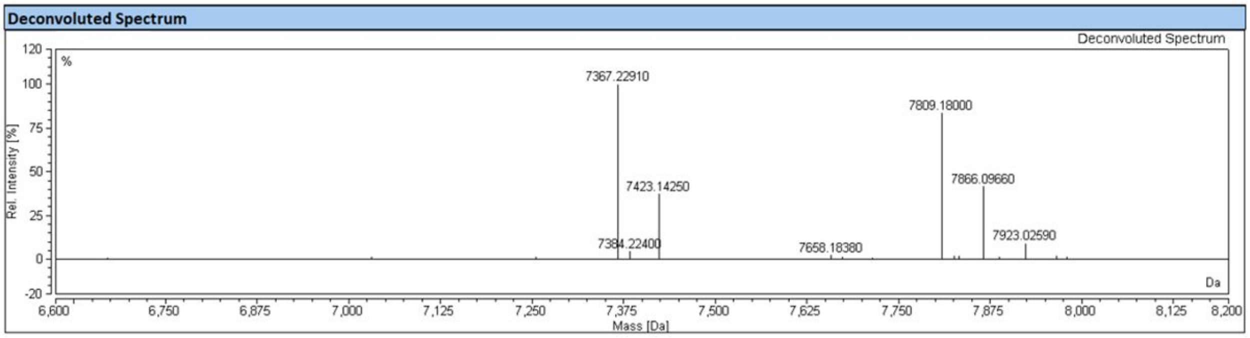

Table S1. The correlation between the GC ratio or theoretical melting temperature and the ratio of intact detected under both polarities for all 4 ARCs. The ratio of intact was calculated based on the peak intensity of intact divided by the sum of that of intact loss of AS and that of intact.

ARC	GC%	Theoretical Melting Temperature (°C)	Peak Intensity under Positive Polarity			Peak Intensity under Negative Polarity		
			Loss of AS	Intact	Ratio of Intact	Loss of AS	Intact	Ratio of Intact
ARC-1	26	49	89400000	19700000	18	24700000	718000	3
ARC-2	35	53	11900000	38600000	76	6590000	1220000	16
ARC-3	39	57	5840000	27600000	83	5790000	4800000	45
ARC-4	48	59	0	35000000	100	859000	3190000	79

Table S2. The qualification parameters evaluated in GMP environment for identity release assay. Following parameters are evaluated, including system suitability, specificity (chromatographic non-interference and identity), repeatability and stability of standard and sample. The theoretical masses of each molecule should include all expected molecular weights of intact DAR and/or intact DAR-AS, as well as the additional masses (+ 18 Da) resulting from linker hydrolysis, since the hydrolysis of the maleimide linker does not compromise the identity of the molecule.

Qualification parameters	Acceptance Criteria
System suitability	• Mass error of the first reference standard must be within 100 ppm of the theoretical mass.<br>• There is no carryover (a peak greater than 10%) in the water injection after the reference standard.
Specificity (chromatographic non-interference and identity)	• The mass difference of each molecule in the portfolio must be above 100 ppm to discriminate each identity.<br>• No interference is observed in the reagent blank or specificity matrix. Interference is defined as a peak in the TIC of the blank or matrix that has the same mass and retention time of the ARC in the reference standard with a peak greater than 10%.<br>• The most abundant observed mass (100% intensity) is within 100 ppm of the theoretical mass.<br>• Report the most abundant observed mass to 1 decimal place.
Repeatability	• RSD of the observed mass must be ≤25% for each set of N=6 replicates.<br>• Report the mean, standard deviation and %RSD to 1 decimal place.
Stability of standard and sample	• Evaluate solution stability for both reference standard and sample (drug substance and drug product).<br>• The observed mass is within 100 ppm for each period, Day 0, Day 3 and Day 7.<br>• Report the latest period with correct mass measurement as the stability period of the solution.
